# Delivery of evidence-based critical care practices across the United Kingdom: A UK-wide multi-site service evaluation in adult units

**DOI:** 10.1177/17511437241293917

**Published:** 2024-11-22

**Authors:** William R Thomson, Zudin Puthucheary, Panayiotis Stavrinou, Dalia Barghouthy, Shreekant Champanerkar, Douglas Findlay, Sarah Gordon, David McWilliams, Kate Tantam, Helen Woodward, Timothy J Stephens

**Affiliations:** 1 Adult Critical Care Unit (ACCU), The Royal London Hospital, Barts Health NHS Trust, London, UK; 2 Critical Care and Perioperative Medicine Research Group, WHRI, Queen Mary University of London, London, UK; 3 Critical Care, University Hospitals Coventry and Warwickshire NHS Trust, Coventry, UK; 4 Critical Care, Grange University Hospital, Cwmbran, UK; 5 Critical Care, Glasgow Royal Infirmary, NHS Greater Glasgow and Clyde, Paisley, UK; 6 Regional ICU, Royal Victoria Hospital, Belfast, UK; 7 SUCCEED Fellow, Queens University Belfast, Belfast, UK; 8 Centre for Care Excellence, Coventry University, Coventry, UK; 9 Critical Care, Derriford Hospital, University Hospitals of Plymouth NHS Trust, Plymouth, UK; 10 University of Plymouth, Faculty of Health, Plymouth, UK; 11 ICNARC Team, The Royal London Hospital, Barts Health NHS Trust, London, UK

**Keywords:** Standards of care, evaluation, ICU liberation bundle, quality of care

## Abstract

**Background:**

The ICU Liberation Bundle was developed to improve outcomes for patients admitted to critical care. Despite a lack of Bundle adoption in the UK, the individual evidence-based practices (EBPs) within the bundle are defined as standards of care by the UK Intensive Care Society. There are limited data on the delivery of these EBPs.

**Objective:**

To evaluate current delivery of the EBPs of the ICU Liberation bundle in a sample of hospitals in the UK National Health Service (NHS) presenting delivery of EBP’s between hospitals, their stability of delivery across multiple weeks and in comparison to US hospitals in the original ICU Liberation Bundle study

**Methods:**

Multi-centre service evaluation, using modified definitions of compliance from the ICU Liberation Bundle study. We sampled six representative units from across the UK; data collection totalled 1116 patient days. Data were analysed using descriptive statistics.

**Results:**

Across all six units, patients received a median of 42.9% (IQR 40%–60%) of all possible bundle EBPs. Unit bundle proportional compliance (number of components completed/eligible number of components) ranged from 40.0% (IQR 28.6%–50.0%) to 71.4% (IQR 57.1%–80.0%). Units completed spontaneous awakening trials most regularly in 80.1% of eligible patients (149/186). Delirium assessments were the least adhered to EBP with only 32.2% (359/1116) of patients receiving at least two validated delirium assessments per day. Full bundle compliance was lower in the UK cohort in comparison to the original trial (4% vs 8%).

**Discussion:**

We identified substantial variation in the delivery of seven evidence-based practices that are considered standards of care in the UK. Variation existed between hospitals and within each hospital over time. These data begin to describe the current state of EBP adherence in a selection of critical care units.

## Introduction

Twenty years of research have led to development of a robust evidence base regarding treatment related harm and its reduction in Critical Care settings.^1–3^ Critically ill patients rely on healthcare professionals to deliver this evidence-based care, including avoidance of unnecessarily prolonged intubation, ventilation and sedation, allowing early mobilisation where appropriate. This is proven to increase survival, ICU-free days and long term function / quality of life.^4–7^ In 2015, the US Society of Critical Care Medicine implemented a new ‘ICU Liberation Bundle’ (the ‘Bundle’) in 68 Critical Care Units. Where the 6 key components were implemented consistently, patient outcomes improved.^8^ Although the Bundle has not been widely adopted in the UK, the individual interventions are standards of care as defined by the Intensive Care Society’s GPICS 2.0^4^

The bundle prescribes six evidence-based practices (EBPs) which form a complex daily intervention for each Critical Care patient. An alphabetised aide-memoire is used: **A**ssess, prevent, and manage pain; **B**oth spontaneous awakening and breathing trials: **C**hoice of Analgesia and Sedation; **D**elirium assess, prevent, and manage; **E**arly mobility and exercise; **F**amily engagement/empowerment.^5^ These should be applicable to every Critical Care patient every day. A Cochrane review confirms that protocolised weaning practices increases both ventilator-free and ICU-free days, with obvious benefit for patient outcomes^5^ A systematic review of 1243 patients found that proactive sedation weaning reduced days spent in both ICU and hospital.^6^ Another systematic review noted that early mobilisation reduces duration of mechanical ventilation, and leads to an increased number of mobile patients at hospital discharge.^7^ There is meta-analysis level evidence for the reduction of delirium in the critical care setting where family engagement is high.^9^ However despite the body of evidence the consistent delivery of EBPs remains challenging, even in the relatively resource rich context of UK Critical Care units.^10,11^ This is driven by the complexity of Critical Care delivery and the interacting factors of high acuity patients, multi- and inter-disciplinary team working, often highly-technical interventions and electronic data systems.^10,12,13^ We also know that inconsistent delivery of other critical care EBPs, such as Low Tidal Volume Ventilation, is widespread and puts patients at risk of worse outcomes. To the authors’ knowledge, no recent data exists that describes delivery of the evidence-based practices from the ICU Liberation Bundle in a United Kingdom context. In Europe, a multi-centre study in Spain demonstrated very low delivery of bundle practices in mechanically ventilated patients.^14^

The objective of this study was to evaluate current delivery of the EBPs of the ICU Liberation bundle in a sample of hospitals in the UK National Health Service (NHS) comparing delivery between hospitals, comparing the stability of delivery across multiple weeks and in comparison to US hospitals in the original ICU Liberation Bundle study.^8^

## Materials and methods

### Study design

A multi-centre, prospective service evaluation of delivery of recommended evidence-based practices (EBPs; ICU Liberation Bundle/UK ICS). The data of interest was *process delivery* rather than patient-specific data; thus, the same patient may have been included multiple times on different data collection days. EBP delivery was assessed across a number of weeks to accurately capture unit wide practice (therefore reflecting embedded policies and procedures) and its variance across weeks (reflecting potentially unreliable processes). Assessment over multiple weeks also mitigated against the risk of capturing only individual clinician preference which may occur in single point prevalence audit.

### Study setting

Six adult general intensive care centres were identified through professional networks representing tertiary Critical Care centres. Effort was made to have a geographical spread of units in all three devolved health systems of Scotland, Wales and Northern Ireland and in different regions in England. The sites represent a mix of major trauma centres, tertiary referral centres and a large district general hospital which serves as a regional Critical Care hub. The sample size was based on the concept of judgement sampling from the evaluation and improvement science literature.^15^ The logic is that it is often more useful to collect a smaller amount of data, over time to capture variation, than to collect a lot of data/sample many sites at a fixed time point for example, a point prevalence study

### Ethics

Using the NHS Health Research Authority (HRA) decision tool this was determined to be a service evaluation of current standards of care and not research. It was registered as such in each participating centre.

### Inclusion and exclusion criteria

All patients being cared for on adult intensive care units were presumed suitable for inclusion. Patients were excluded if they had been in Critical Care for less than 24 h or were deemed to be for active end of life care. Given the need to collect data at the patient’s bedside patients were allowed to be pragmatically excluded if the data collection team could not access the bedside (communicable disease requiring isolation, acute patient deterioration).

### Data collection

Sites collected data at least once a week with an aim of 6–10 weeks of data collection, to assess process variation over time. Each patient in a bedspace was assessed as a single episode. Given that some patients had a prolonged ITU admission data could be collected on the same patient on more than 1 day. This is intentional in order to allow examination of the processes as opposed to individual patient factors. Each assessment period is referred to as a patient day that is, one patient in an ITU bed for a complete 0800-0800 period. Data points were collected for each patient day for the preceding 24 h charting period using minimally adapted operational definitions of bundle performance from the original ICU Liberation bundle (Table 1).^8^ Sites were instructed to collect patient data from their whole unit each time they undertook a data collection session. Individual data collection days may vary given unit occupancy, and pragmatic exclusion criteria. All patients who were present on the ITU were included in the data set (there are two incidences of a child <16 being included in the data set).

**Table 1.** Operational definitions for bundle compliance. Adapted from Pun et al.^8^

Bundle element	Delivery eligibility	Measurement definition
A – pain assessment	All days	⩾6 pain assessments using a valid and reliable instrument/24 h
B1 – spontaneous awakening trial (SAT)	Only days when: patient received continuous IV sedation (Propofol, Midazolam, Dexmedetomidine, Clonidine); and not contraindicated according to local protocol	A documented attempt to cease IV sedation or reduction by at least 50% to allow the patient to awaken (even if unsuccessful)
B2 – spontaneous breathing trial (SBT)	Only days when: patient on mechanical ventilatory support (Continuous positive pressure via Endotracheal tube or Tracheostomy); and no contraindication to SBT (according to local protocol) or pre agreed ventilator weaning plan that would override need for SBT	A documented attempt to reduce ventilatory support to minimal ventilatory settings (e.g. PS of 5–7 cmH_2_O and 1–5 cmH_2_0 PEEP, according to local protocol) to allow the patient to breathe for themselves under load (even if unsuccessful).
C – sedation scoring	All patient days	⩾6 agitation-sedation assessments using a valid and reliable instrument/24 h
D – delirium assessment	All days	⩾2 delirium assessments using a valid and reliable instrument/24 h
E – early mobilisation	All days, unless mobilisation contraindicated	Mobilisation activities that were higher than active range of motion (i.e. sitting on edge of bed, standing at side of bed, walking to bedside chair, marching in place, walking in room or hall)/24 h
F – family involvement	All Days	Presence of personal effects (family pictures, own bedding etc.), up to date personal information board, completion of a patient diary/24 h

For elements A, C, D & E, simple counts were made of the number of observations/interventions made during the preceding 24-h observation period (0800-0800 or 0000-2400 depending on local charting practices). For elements B1 and B2, an assessment was made of whether the patient was receiving continuous IV sedation or mechanical ventilation at 1000 the day prior to data collection. Conventionally, all relevant members of the multi-disciplinary team will have undertaken their initial daily patient assessment by this time – however many ward rounds will just be starting. An assessment was then made by the data collection team (all staff with sufficient Critical Care experience) of whether the patient was eligible for an SAT/SBT the day prior from the available data depending on local protocol, and whether that was then enacted. In units where SBTs and SATs were protocolised, a documented contra-indication would not be recorded as non-compliance. Likewise, if a patient had a collaborative weaning plan that would render an SBT potentially counter-productive, this would not be recorded as non-compliance.

For element F a different definition was utilised than the original US study. The original definition for family involvement is ‘a family member/significant other was educated on the ABCDEF bundle and/or participated in at least one of the following: rounds; conference; plan of care; or ABCDEF bundle related care’.^8^ Families are not routinely involved in medical interventions or routine decision-making in UK practice and therefore more pragmatic definitions were utilised. GPICS 2.0 recommends the use of patient diaries, ‘This is Me’ white boards and of patient’s personal items as some ways of engaging families. These are easily measurable due to their physical presence at the bedside. When collating data the collection team would note the presence of these as evidence of ‘family engagement’.

Every patient was eligible for between four and seven interventions depending on their clinical condition that is, a patient with severe ARDS receiving a neuromuscular blockade would only be eligible for four interventions given that mobilisation, SBT and SAT would all be contraindicated, whereas the same patient a few days later if improving could be eligible for all seven once the clinical condition was stable enough to proceed.

### Data analysis

Data from each hospital site were collated and supplied for central processing. Data were cleaned and descriptive statistics were processed on Microsoft Excel v16.61.1. For elements A, C, D and F, every patient was eligible to receive the interventions every day. Of these, the frequency of those that received the intervention was calculated for each site and for the cohort. For elements B1, B2 and E, lack of ventilatory support/IV sedation and ineligibility for SAT, SBT or mobilisation meant a differing denominator was needed for each bundle element due to differing eligibility. The number of target EBPs that each patient was eligible to receive was calculated and of those, the number they actually received used to calculate bundle compliance per patient. From this the median percentage compliance of EBPs that patients were eligible for, versus which they received were calculated for each unit each week, each unit for the whole duration and for the cohort. This produced a breakdown of the overall percentage of eligible patients receiving each individual element of the bundle. Boxplots were generated for each unit to describe the distribution of percentage compliance for each week of data collected. Boxplots were generated for each unit to describe both the median and overall distribution of percentage compliance for each week of data collected. As this was a conducted as service evaluation, we specifically chose to not undertake any inferential or comparative statistical analysis of differences in intervention delivery. However, it is accepted in the improvement science literature that delivery of a standard of care process should not be <80% and ideally should be >90 or >95%, depending on the level of clinical importance of that process.^16,17^ Using these thresholds allows some simple, yet useful, visual inspection of data over time to make judgements about delivery of standard of care processes.

## Results

Data were collected for 1116 patient days nationally across six sites. In keeping with intensive care populations more generally, there was a higher proportion of males than females. The mean age was 56 years of age (see Table 2).

**Table 2.** Demographic and characteristic information for each site.

	I	II	III	IV	V	VI	All units
Unit character	MTC	MTC	Large DGH	MTC	Tertiary Centre	MTC	
Median age	57	53	62	59	59	59	58
Male sex	151 (57.4%)	111 (59.7%)	122 (58.9%)	94 (53.4%)	53 (59.6%)	121 (62.1%)	651 (58.3%)
Number IV sedation	113 (43.0%)	81 (43.5%)	52 (25.1%)	55 (31.3%)	14 (15.7%)	95 (48.7%)	410 (36.7%)
Number mechanically ventilated	123 (46.8%)	146 (78.5%)	67 (32.4%)	92 (52.3%)	30 (33.7%)	137 (70.3%)	595 (53.3%)

MTC: major trauma centre; DGH: district general hospital.

### Individual practices delivered

#### Element A – pain scores

Across sites, the frequency of delivery of the pain element of the bundle, 6 or more assessments of pain in each period, was 54.0% (603/1116). There were significant variations in compliance between sites on the delivery of this element (range: 9.1%–99.4%) (see Table 3).

**Table 3.** Bundle element standard compliance by site (I–VI) and in aggregate (all units). Please see Table 1. for operational definitions of compliance.

	Unit code	I	II	III	IV	V	VI	All units
	Patient days assessed	263	186	207	176	89	195	1116
A – Pain assessment	Number compliant to standard	24 (9.1%)	116 (62.4%)	141 (68.1%)	175 (99.4%)	43 (48.3%)	104 (53.3%)	603 (54.0%)
B1 – SAT	Number IV sedation	113 (43.0%)	81 (43.5%)	52 (25.1%)	55 (31.3%)	14 (15.7%)	95 (48.7%)	410 (36.7%)
	SAT eligible	37 (14.1%)	33 (17.7%)	21 (10.1%)	36 (20.5%)	11 (12.4%)	48 (24.6%)	186 (16.7%)
	Number compliant to standard	28 (75.7%)	22 (66.7%)	18 (85.7%)	25 (69.4%)	10 (90.9%)	46 (95.8%)	149 (80.1%)
B2 – SBT	Number mechanically ventilated	123 (46.8%)	146 (78.5%)	67 (32.4%)	92 (52.3%)	30 (33.7%)	137 (70.3%)	595 (53.3%)
	Eligible for SBT	8 (3.0%)	40 (21.5%)	21 (10.1%)	23 (13.1%)	6 (6.7%)	32 (16.4%)	130 (11.6%)
	Number compliant to standard	7 (87.5%)	19 (47.5%)	16 (76.2%)	15 (65.2%)	6 (100%)	23 (71.9%)	86 (66.2%)
C – Agitation assessment	Number compliant to standard	214 (81.4%)	155 (83.3%)	127 (61.4%)	176 (100%)	87 (97.8%)	102 (52.3%)	861 (77.2%)
D – Delirium assessment	Number compliant to standard	7 (2.7%)	106 (57.0%)	9 (4.3%)	135 (76.7%)	70 (78.7%)	32 (16.4%)	359 (32.2%)
E – Early mobilisation	Number compliant to standard	82 (31.2%)	60 (32.3%)	125 (60.4%)	78 (44.3%)	55 (61.8%)	61 (31.1%)	461 (41.3%)
F – Family involvement	Number compliant to standard	239 (90.9%)	44 (23.7%)	205 (99.0%)	117 (66.5%)	39 (43.8%)	91 (46.7%)	735 (65.9%)
Overall bundle compliance	Median% (IQR)	40.0% (28.6%–50%)	50.0% (33.3%–57.1%)	57.1% (40.0%–66.7%)	71.4% (57.1%–83.3%)	60.0% (57.1%–80%)	40%–0% (28.6%–42.9%)	42.9% (40.0%–60.0%)

#### Element B1 and B2 – SAT and SBT

There was inter-site variation regarding the proportion of patients receiving continuous sedation (15.7%–48.7%), and mechanical ventilation (33.7%–78.5%). 80.1% (149/186) adherence was seen in the SAT bundle element, (range: 66.7%–95.8%). Regarding SBTs in patients who were eligible, adherence to the bundle element was 66.2% (86/130) (range: 47.5%–100%).

#### Element C – sedation/agitation scoring

For element C, 77.2% (861/1116) of patients in the cohort had sedation/agitation scoring at least six times/day (range: 52.3%–100%).

#### D – delirium assessment

For element D there was widespread heterogeneity between sites’ delivery. Validated delirium assessment (⩾2 assessments per day) was the least delivered bundle element, with completion in 32.2% of patient days across all sites (range: 2.7%–77.2%).

#### Element E – early mobilisation

There was variation in the degree of early mobilisation between sites (range: 31.2%–61.8%, sitting on bed edge or better). Overall early mobilisation was conducted in 41.31% (461/1116) of patients.

#### Element F – family engagement

The family engagement interventions demonstrated the largest variation between sites (range: 23.7%–99.0%). The sites over 90% provided patient diaries to all patients as part of an admission bundle. Across the cohort 65.9% (735/1116) demonstrated evidence of friends and family engagement or personalisation of care.

### Total practice delivery per day

Bundle compliance was collated for each patient day. A median was calculated for the cohort overall, and for individual sites. Overall bundle element completion across sites was a median of 42.9% (IQR 40%–60%). Sites ranged individually from a median compliance of 40% (IQR 28.6%–50%) to 71.4% (IQR 57.1%–83.3%) (see Table 3).

**Figure 1.**
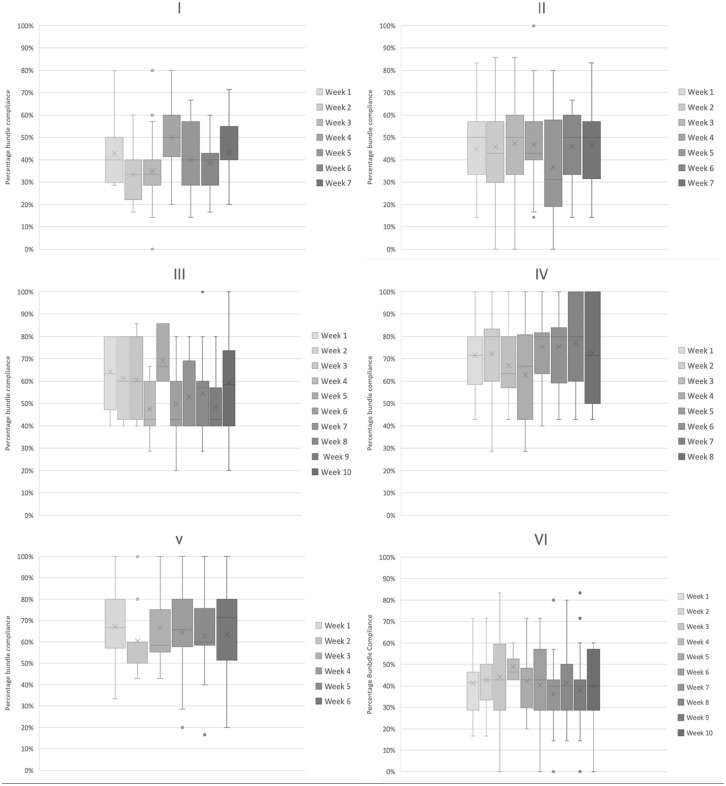
Box plots of weekly compliance with (a–f) Bundle elements by site (I–VI), demonstrating median, IQR, range and outliers for overall bundle compliance.

Figure 1 shows the distribution of compliance with practice delivery each week. Even without comparative statistics visual inspection of the charts can be useful to infer both the stability (or variation) of delivery egarding variation, visual inspection of the charts suggests that three sites have a more consistent compliance week on week (Sites II, IV and to a lesser extent V).This may indicate a more stable care delivery process for the EBPs that in theory should be delivered to every patient, every day and so should not be subject to much variation. Regarding element delivery, most of these EBP’s are GPICS standards of care (albeit with some differences in definitions as outlined in the Discussion section) and as such one would expect >95% delivery. When evaluating processes ⩾80% completion can be considered the minimum evidence of a reliable underlying process.^17,18^ Even allowing for this lower threshold only once site (IV) achieved ⩾80% median compliance on any week (4/8 weeks assessed). Overall, compliance data from this cohort compared to the cohort in the original ICU Liberation Bundle^8^ study is shown in Table 4.

**Table 4.** Comparison of UK and US adherence to A-F bundle EBPs. UK data compliant to definitions in Table 1. US Data from Supplemental File of Pun et al.^8^

	UK	US
Patient days	1116	49,018
A	54%	77%
Number IV sedation	37%	45%
B1 – % compliant	80%	34%
Number mechanically ventilated	53%	50%
B2 – % compliant	66%	36%
C – % compliant	77%	59%
D – % compliant	32%	56%
E – % compliant	41%	29%
F – % compliant	66%	63%
Full compliance	4%	8%

## Discussion

In this study, using a median of 80% bundle compliance as an acceptable threshold, we have identified substantial variation in the delivery of seven evidence-based practices that were utilised in the initial Liberation Bundle study. This variation was seen between all six hospitals, but also appeared present within each hospital over time. Improvement science theory suggests that if the variation we describe exists in these six, totally separate and disparate critical care units, it is highly likely that many other UK units will also have similar variation and performance levels.^15^

Overall, sedation assessment and SATs were the most frequently delivered practices, whereas delirium assessments and early mobilisation were the least delivered. Comparison with USA data identified differing areas of strength and weakness regarding delivery of the practices, but it is notable that the UK performance has been achieved outside of a large-scale study, major campaign or improvement programme. The Bundle standards as noted in the limitations sometimes represent a more stringent standard than GPICS, however moving from an acceptable performance baseline (as our study data indicate) to excellent performance poses different, more complex challenges, than if starting from a lower baseline.^19^ Given that these practices are identified as GPICS standards of care, there is the suggestion that further improvements can, and need to be, achieved.

Failing to deliver care that is known to provide patient benefit is described as the ‘know-do’ gap.^20,21^ Other recent multi-centre studies have identified know-do gaps in several critical care areas. These include adherence to lung protective ventilation, daily assessment of patients’ readiness for cessation of mechanical ventilation^22–24^; the application of sedation interruptions^25–27^; and early mobilisation in critical care UK data indicating particularly low levels.^28,29^ It is well known that the translation of evidence into practice takes much longer than clinicians want (or that patients should demand). Yet solutions to this pervasive problem are not particularly forthcoming. Critical care teams (nurses, doctors and therapists) are best placed to identify local ‘know-do’ gaps, but they are experts in clinical, not implementation science and few have the skills to develop effective and local context-appropriate solutions.^30,31^ Collaborations both locally and also at a national level with implementation scientists is one solution for implementation capacity building within critical care units.^32^ Data is key to creating the situational awareness needed to guide implementation efforts. A recent systematic review of electronic Audit & Feedback strategies identified availability of real-time data for feedback as a key factor in achieving clinical improvement.^33^ However, data collection can be a major distraction for clinicians, stalling the necessary work to improve care.^34,35^ Relevant critical care data exists within local Electronic Health Record (EHR) systems but remains difficult to access across much of the NHS.^33,36^ Many clinicians do not find EHRs user-friendly, even for daily practice.^37^ Four of the six units in this evaluation used EHR (as opposed to paper) charting but only in two of these was it stated that this actually reduced the time and labour burden of data collection. Issues such as data-warehousing, and system designs that lack easily modified reporting functions, further hamper use of EHR data for implementation efforts. Data collaborations such as the Critical Care Health Informatics Collaborative (CCHIC) are commendable but such efforts to pool data need to be paired with better, widespread local data availability. Alongside implementation science strategies to routinise standard care, we urgently need solutions to facilitate use of EHR data for the monitoring of EBPs such as those in this study.

We propose two main implications for practice and the critical care community from this study. First, now we have the evidence of variation we suggest other units consider audit or monitoring of their EHR data to assess local delivery of these EBPs. To address the identified adherence issues, we suggest some qualitative/mixed methods work will be necessary to understand the causes of such variation and the various successes and challenges faced by units when implementing these standards of care. This will provide some empirical evidence that can be used to support both this cohort and similar units across the country who are likely to face at least some of the same influences on implementation/non-implementation. This requires a deep understanding of local Critical Care unit contexts, rigorous monitoring which requires easy access to local performance data, and implementation strategies that promote sustained improvement.^38,39^

### Strengths

To our knowledge this work provides the first snapshot of A–F bundle adherence practices from multiple UK sites. It is likewise mostly comparable with a US study, using similar definitions with only minimal adaptions. The authors represent a spread of hospitals of differing sizes, and location (including from all four constituent parts of the UK to reflect the devolution of health care in the UK). The definitions used are pragmatic and represent de facto clinical activity. We note the divergence between the GPICS standards and the Liberation trial definitions. For some EPBs GPICS gives a recommendation of a practice without specifying a frequency (i.e. Pain assessment). The pragmatic standards utilised in this audit attempt to homogenise two pieces of guidance, therefore allowing a working audit tool that reflects an aspirational standard for UK practice. This allows international comparisons whilst reflecting guidance set forward by the Intensive Care Society.

### Limitations

General weaknesses of this type of study include its observational nature, and the lack of outcomes tied to patient bundle compliance. It was run under service evaluation guidelines and therefore findings cannot be generalisable but reflective of the centres in which it took place. Likewise, the number of patients analysed was small when compared to the US data set. Heterogenous populations at different sites are noted with levels of dependence of patients between units which may have an influence on adherence. Inter site heterogeneity for SBTs may be explained by differences in proportions of mechanically ventilated patients and SBT eligibility numbers. Sites were urged to use local protocols to determine eligibility which may exacerbate these differences. Some level of interpretation was required by the data collectors themselves that is, did this person have a SAT or not from chart data. This may have led to a more generous or less assessment of adherence to stated standards. Some data were not available to teams that is, PRN drug charts for IV haloperidol which would have brought operational definitions more into line with the US study. The included centres represent departments of larger critical care centres and therefore may not be representative of district general hospitals and smaller centres. Units collated data on weekdays, given the data was collated 24 h for the preceding data does not represent activity on Fridays or Saturdays. Given the challenges some units have regarding the provision of AHP and medical teams over weekend this may not be reflected in entirety.

As noted in the strengths of this work there is divergence between the GPICS standards and those utilised in the Bundle. For example GPICS states ‘All patients must be screened daily for evidence of delirium using a validated method such as the Confusion Assessment Method for the ICU (CAM-ICU) or the Intensive Care Delirium Screening Checklist (ICDSC)’ whereas the A–F bundle requires twice daily assessment. For agitation assessments GPICS only recommends scoring patients receiving sedation whereas the liberation bundle recommends this for all patients. GPICS states that ‘The intensive care physiotherapy service or, where appropriate, as part of the MDT, should have robust and evidence-based clinical guidelines/standard operating procedures surrounding . . . rehabilitation interventions including early mobilisation of patients in intensive care’ without further elucidating the frequency or eligibility.^4^ This effectively means that some assessments in this service evaluation holds units to higher account than is recommended by current service provision guidance.

## Conclusions

Despite being considered standards of care in the UK, we found substantial headroom to increase the reliable delivery of key evidence-based practices that are known to improve patient outcomes.
